# A signal-based method for finding driver modules of breast cancer metastasis to the lung

**DOI:** 10.1038/s41598-017-09951-2

**Published:** 2017-08-30

**Authors:** Gaibo Yan, Vicky Chen, Xinghua Lu, Songjian Lu

**Affiliations:** 1 Department of Biomedical Informatics, University of Pittsburgh, Pittsburgh, PA USA; 2 Center for Causal Discovery, University of Pittsburgh, Pittsburgh, PA USA

## Abstract

Tumor metastasis is mainly caused by somatic genomic alterations (SGAs) that perturb pathways regulating metastasis-relevant activities and thus help the primary tumor to adapt to the new microenvironment. Identifying drivers of metastasis, i.e. SGAs, sheds light on the metastasis mechanism and provides guidance for targeted therapy. In this paper, we introduce a novel method to search for SGAs driving breast cancer metastasis to the lung. First, we search for transcriptomic modules with genes that are differentially expressed in breast cell lines with strong metastatic activities to the lung and co-expressed in a large number of breast tumors. Then, for each transcriptomic module, we search for a set of SGA genes (driver modules) such that genes in each driver module carry a common signal regulating the transcriptomic module. Evaluations indicate that many genes in driver modules are indeed related to metastasis, and our methods have identified many new driver candidates. We further choose two novel metastatic driver genes, *BCL2L11* and *CDH9*, for *in vitro* verification. The wound healing assay reveals that inhibiting either *BCL2L11* or *CDH9* will enhance the migration of cell lines, which provides evidence that these two genes are suppressors of tumor metastasis.

## Introduction

Breast cancer is the most common type of cancer in women, accounting for about one quarter of all cancer cases^1^. Metastasis takes place in the late stages of cancer development and is the major cause of mortality in patients with breast cancer and other solid tumors^2, 3^. As with other cancer processes, such as resisting cell death and sustaining cell proliferation, cancer metastasis is mainly caused by somatic genomic alterations (SGAs) such as somatic mutations and copy number alterations that perturb signaling pathways regulating metastasis-relevant activities, including fiber formation and focal adhesion, epithelial–mesenchymal transition, differentiation and morphogenesis, and invasion^4^. Somatic mutations and copy number alterations of a gene may change its 3D structure and the concentration of its gene product – protein, respectively. Hence, if the protein is a signaling protein, then the signal it carries will be disturbed. The signal perturbation causes abnormal expression of genes regulated by the signal, thus altering the behavior of cells, such as turning normal cells into cancerous ones or enabling tumor cell invasion and metastasis.

An important way to study the cancer metastasis mechanism is to find gene subsets or gene signatures that are involved in metastasis-relevant activities by contrasting the expression data of cancer cells with and without metastasis. For example, Minn *et al*. compared gene express profiles of MDA-MB-231 subpopulations with different degrees of lung metastatic activities to identify a set of genes that likely mediates breast cancer metastasis to the lung^2^. In another study, Bos *et al*. isolated subpopulations with strong brain metastatic activity from parental CN34 and MDA-MB-231 cell lines and then found genes that mediate the metastasis of breast cancer to the brain by comparing expression data of cell lines with different strengths of brain metastatic activity^5^. Harrell *et al*. found the gene signatures of breast metastasis to the brain, lung and liver by comparing expression data of primary tumors and their matched metastases or sets of synchronous metastases from the same patients^6^. However, while such studies shed light on the genes involved in the molecular mechanisms of metastasis, they do not necessarily reveal the drivers or pathways that originally activate the process of metastasis.

From the point of view of targeted therapy or precision medicine, a more important task in cancer metastasis research is to search for drivers, i.e. SGAs that initiate cancer metastatic progression, where those drivers provide ideal candidates for targeted therapy. There are abundant publications in this line of research. For example, Wagenblast *et al*. found that the genes *SERPINE2* and *SLPI* are drivers of metastasis by exploring the impact of enforced expression of these two genes on metastasis^7^. Julien *et al*. investigated the role of gene *PTP1B* in mammary tumorigenesis by inhibiting it *in vivo*. They found that the rate of tumor development, number of tumors and lung metastases were significantly reduced in *NDL2* transgenic mice both in *PTPN1*-/- mice and in *PTP1B* inhibitor treated mice^8^. Xue *et al*. showed that loss of the gene *PAR3* promoted breast cancer metastasis *in vitro* and *in vivo* ^9^. Tan *et al*. found that the *RANKL*-*RANK* signal regulated the cancer metastasis of T cells^10^. All of the above examples used wet-lab techniques to search for drivers of metastasis. Currently, a large amount of gene expression, somatic mutation, copy number alteration etc. data for cell lines with metastasis^2, 5^, breast tumors with relapse in the brain, lung, liver etc.^5, 6, 11^ is becoming available. Hence, using computational methods to search for drivers of metastasis has become feasible. Computational methods can provide short lists of candidates that are very likely to be drivers of metastasis, which can then be verified by biologists, thereby greatly speeding up the research into understanding the disease mechanism of cancer metastasis.

Drivers of metastasis can be used as candidates for targeted therapy. However, not all drivers are “druggable”^12, 13^; furthermore, as FDA-approved drugs for targeted therapy is limited, only a small number of drivers in tumors can be directly treated by approved agents ^14^. One way to address this problem is to find out what drivers are on the same pathway. Then if a driver in a tumor cannot be drugged directly, we can target other genes/proteins that carry the same signal carried by the “undruggable” driver. For example, human *RAS* genes are a notorious oncogene family, where no effective inhibitor has reached the clinic despite more than 30 years of effort^13^. One current major effort for anti-*RAS* drug discovery is aiming to block the activities of proteins that are components of the signaling pathway downstream of *RAS* ^13^. In this study, we will develop a new computational framework that not only finds drivers of breast cancer metastasis to the lung, but also groups drivers carrying common signals together.

The *de novo* discovery of pathways underlying specific biological processes, e.g., metastasis, remains a challenging task. First, few studies of cancer metastasis collect omics data that reflect different aspects of the metastatic process. For example, while collecting only genomic data from metastasized tumors does reveal which genomic alterations are specifically driving metastasis, identifying differentially expressed genes (DEGs) does not reveal the driver gene or pathway that regulates the DEGs. In this study, we designed a framework that integrates the results of transcriptomic studies of metastasis with existing large-scale comprehensive omics data to enable a search for the SGAs driving expression of genes involved in metastasis. A second challenge to *de novo* discovery of such pathways is there are few well-established computation methods to search for SGA drivers that causally regulate the expression of a set of genes. Therefore, in this study, we developed a novel *de novo* computational framework to search for SGAs driving breast cancer metastasis to the lung.

In this framework, first, we found out what genes were differentially expressed in a subpopulation of breast cell lines with strong metastatic activity to the lung. Then, we used differentially expressed genes to search for gene transcriptomic modules such that the genes in each transcriptomic module are most likely to be regulated by a common signal. Next, we used the expression status of each transcriptomic module as the readout of a signal to search for genes whose SGA events have strong information with respect to the expression status of transcriptomic modules. Finally, we chose two genes from our results for *in vitro* verification (Please refer to the overall scheme in the METHODS section).

## Results

### Gene transcriptomic modules

**Figure 1:**
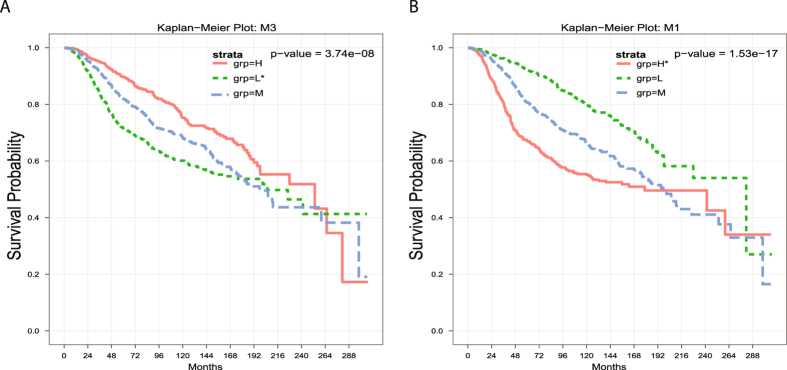
The expression statuses of gene transcriptomic modules have impact on patients’ survival. H, M, L represents tumor group with high, middle, low expression of the transcriptomic module, respectively. A “*” after H/L signifies that the module is up-/down-regulated in high metastatic cell lines.

We first found the differentially expressed genes (DEGs) by comparing the MDA-MB-231 parental cell line or its subpopulations that have relatively weaker lung metastasis activities with other subpopulations that have relatively stronger lung metastasis activities. Then the number of DEGs was further refined by excluding genes that were up-regulated in cell lines and down-regulated in lung tumors (TCGA) or down-regulated in cell lines and up-regulated in lung tumors. Next, we searched for gene transcriptomic modules from DEGs such that the genes in each gene transcriptomic module were co-expressed in a large number of TCGA breast tumors and also regulated by a common transcription factor (refer to METHODS for details). Hence, it is most likely that the genes in each gene transcriptomic module are regulated by a common signal. For each gene transcriptomic module, we obtained two tumor subsets *T*_*abn*_ and *T*_*nor*_, where *T*_*abn*_ included BRCA tumors that had abnormal expressions of genes in the module and tumors in *T*_*nor*_ had expressions of genes in the module similar to normal samples. We found 21 gene transcriptomic modules. We checked if the expression statuses of the transcriptomic modules impacted patients’ clinical outcomes. As the TCGA BRCA clinical data did not have enough death events, we used METABRIC^15^ data to evaluate the impact of our gene transcriptomic modules on patients’ survival. The results showed that 11 of 21 modules had significant impact (both *p*-value and *q*-value less than 0.05) on patient survival (refer to Fig. 1 and Fig. S1). Moreover, usually, if a transcriptomic module was down-regulated in high metastatic cell lines, then patients with a low expression of the transcriptomic module had a worse clinical outcome (refer to Fig. 1A). On the other hand, if a transcriptomic module was up-regulated in high metastatic cell lines, then patients with a high expression of the transcriptomic module had a worse clinical outcome (refer to Fig. 1B). Hence, our transcriptomic modules are biologically meaningful as cancer metastasis is the major cause of mortality in breast cancer patients^2, 3^.

### Driver modules

We assume that each gene transcriptomic module is regulated by a common signal. The abnormal expression of genes in a gene transcriptomic module in a tumor is caused by at least one SGA event that perturbs the common signal regulating the module. The major goal of the project is identifying drivers carrying common signals, where drivers provide candidates for targeted therapy; in the case where a driver *g* in a tumor is not “druggable”, we can instead target one of the other drivers that carry the same signal that driver *g* does. For this purpose, we developed a signal-based method that searched for sets of genes such that their SGA events have strong information with respect to common signals (refer to METHODS for detail).

**Figure 2:**
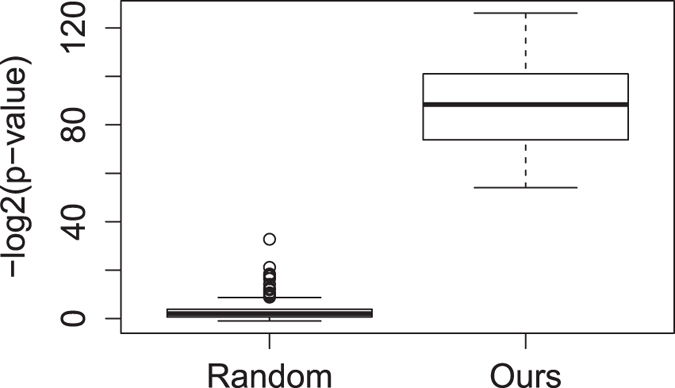
Enrichment *p*-values for random and our driver modules. SGA events of genes in our driver modules are greatly enriched among tumors in *T*_*abn*_s.

For each gene transcriptomic module, we obtained two tumor subsets *T*_*abn*_ and *T*_*nor*_, where we suppose that tumors in *T*_*abn*_ are highly likely to have SGA events that perturb the common signal regulating the gene transcriptomic module while tumors in *T*_*nor*_ are less likely to have SGAs that perturb this common signal. Hence, if a set of genes are drivers that carry the common signal regulating a gene transcriptomic module, then the SGA events of those genes should mostly happen among tumors of *T*_*abn*_ and seldom occur among tumors in *T*_*nor*_. Corresponding to the 21 gene transcriptomic modules, we obtained 21 driver modules. Each driver module has six genes. Our results show that SGA events of genes in all driver modules are very enriched among the corresponding *T*_*abn*_s. The *p*-values calculated from a hypergeometric distribution are between 1.04E-38 and 5.47E-17, which is much better than driver modules obtained from random selection (refer to Fig. 2). These results reveal that SGA events of genes in driver modules have strong information with respect to corresponding common signals.

### Evaluate driver modules via literature search

A literature search revealed that SGA events of many genes in our driver modules were related to cancer metastasis, cell invasion or migration, where many of those SGA-metastasis associations were obtained from *in vitro* or *in vivo* verifications. We kept references about cell invasion and migration as they are important processes of metastasis.

**Figure 3:**
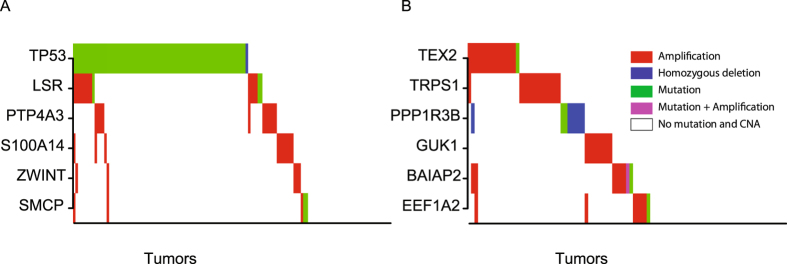
SGA pattern of driver modules. (**A**) SGA pattern of module 1. (**B**) SGA pattern of module 18.

One example is driver module 1, which includes the genes *LSR*, *PTP4A3*, *S100A14*, *SMCP*, *TP53*, and *ZWINT*, where *TP53* was either mutated or deleted; *SMCP* had both mutations and amplifications; the remaining four genes were mainly amplified (refer to Fig. 3A). García *et al*. found that *LSR* (*LISCH7*) mRNA in plasma was significantly associated with lymph node metastasis and with vascular invasion, and they thought that the up-regulation of the gene promotes the development of metastasis^16^. Bayat *et al*. reported that *CD177*(+) neutrophils migrated significantly faster through HUVECs expressing *LSR* in *in vitro* transendothelial migration experiments^17^. Guzinska-Ustymowicz *et al*. found that high expression of *PTP4A3* was associated with lymph node metastasis from colorectal carcinoma^18^. Laurent *et al*. claimed that high *PTP4A3* phosphatase expression correlates with metastatic risk and expressed the belief that *PTP4A3* plays a causal role in the development of metastases in uveal melanoma^19^. Zimmerman *et al*. verified that a knock-out of *PTP4A3* decreases migration of endothelial cells by *in vitro* wound healing assay^20^. Cho *et al*. demonstrated that *S100A14* promotes cell migration and invasion using a wound healing assay and Martrigel invasion assay *in vitro* ^21^.

Zhao *et al*. claimed that *S100A14* promotes the growth and metastasis of hepatocellular carcinoma. Their claim was based on the wound healing assay and Matrigel transwell assay, which verified that *S100A14* silencing suppressed cell migration and invasion while overexpression of *S100A14* promoted migration and invasion of hepatocellular carcinoma cells. They also validated *in vivo* that inhibiting *S100A14* significantly reduced lung metastasis in mouse models^22^. Takahashi *et al*. explored that *SMCP* might be a novel marker of CSC/CIC cells, which are highly responsible for disease recurrence after treatments and for distant metastasis^23^. *TP53* is a well-known tumor suppressor that is associated with cell cycle arrest, apoptosis, and DNA repair etc. that also regulates cancer metastasis. Patricia *et al*. stated that mutant p53 proteins promote invasion, metastasis, proliferation and cell survival^24^. Adorno *et al*. validated with wound healing assays that mutant-p53 (p53 R175H) promoted the migration of H1299 cells and validated with transwell assays that mutant-p53 is required for TGFβ-driven invasion and metastasis in breast cancer MDA-MB-231 cells^25^. Weissmueller *et al*. demonstrated that mutant p53 promoted migration and metastasis *in vitro* and *in vivo* ^26^. Hence, five of six genes in this module have been verified to be drivers of metastasis.

Another example is driver module 18, which includes the genes *BAIAP2*, *EEF1A2*, *GUK1*, *PPP1R3B*, *TEX2*, and *TRPS1*. Except for gene *PPP1R3B*, which was mainly mutated or deleted, the other five genes were mainly amplified among TCGA breast tumors with abnormal expression of genes in gene transcriptomic module 18 (refer to Fig. 3B). Funato *et al*. stated that *BAIAP2* (*IRSP53*) is important for the metastatic behavior of malignant tumor cells^27^. In^28^, Hoeppner *et al*. said that shRNA to *BAIAP2* inhibited podocyte migration. Kawamura *et al*. verified that siRNA *EEF1A2* inhibited cell migration significantly in LCSC#1 cell lines^29^. Xu *et al*. used the Matrigel transwell assay to evaluate the invasive capacities of cell lines with different expression levels of the *EEF1A2* gene and found that more invasive cell lines have higher *EEF1A2* expression. They also found that siRNA *EEF1A2* suppressed cell invasion and migration in both a Matrigel transwell assay and wound healing assay. Hence they concluded that *EEF1A2* promotes cell migration, invasion and metastasis in pancreatic cancer^30^. De Rocha *et al*. found that *GUK1* was differentially expressed in metastatic pituitary carcinoma^31^. Hu *et al*. found that over-expression of *TRPS1* promoted HUVEC migration significantly and knockdown of *TRPS1* decreased this migration ability^32^. Hong *et al*. stated that high *TRPS1* expression was significantly associated with positive lymph node metastasis^33^. Therefore, the SGA events of four genes in this module have been verified to be associated with cancer metastasis. We could not find references that associated genes *PPP1R3B* and *TEX2* with metastasis, migration, or invasion directly. However, we found that Lu *et al*. claimed that the mutated *PPP1R3B* peptide represents the immunodominant epitope^34^ and Hayashida *et al*. claimed that *PPP1R3* gene alterations correlated with lymph node and liver metastases^35^, which provides evidence of association between *PPP1R3B* gene and metastasis.

Further study showed that in our 21 driver modules, two modules have five genes and three modules have four genes related to metastasis, respectively. Another four modules have three genes and nine modules have two genes related to metastasis, respectively. Finally, the remaining three modules have one gene related to metastasis (refer to Table S1). Hence, our driver modules are biologically meaningful.

We also used a commercial software – “Ingenuity Pathway Analysis (IPA)” to check the functional coherence of our driver modules. Results showed that many of our driver modules were significantly (*p*-value were much less than 0.05) related to certain functions related to metastasis activities. For example, four genes, *ATG7*, *ERBB2*, *NME1*, *ODF2* in driver module 2 were associated with the formation of cellular protrusions; three genes, *GNA13*, *GRHL2*, *TNFRSF10B* in driver module 6 were related to the morphogenesis of fibroblast; and three genes, *ATAD5*, *CLCF1*, *PIK3CA* in driver module 8 were associated with the proliferation of B lymphocytes.

### Verify new drivers of metastasis through *in vitro* wound healing assay

We further chose two genes, *BCL2L11* and *CDH9*, from our driver modules to verify if they are drivers of metastasis. To the best of our knowledge, no experiments have been performed to verify the relation of these two genes to cancer metastasis. The protein encoded by *BCL2L11* belongs to the BCL-2 protein family and has been shown to interact with other members of the BCL-2 protein family and to act as an apoptotic activator. *BCL2L11* is also called *BIM*. Much research about this gene has been focused on the association of this gene with cell death or apoptosis^36–38^. *BIM* can abrogate the functions of all anti-apoptotic proteins in the Bcl-2 family, Mcl-1 and A1^39^. There exists research that mentioned the association of *BIM* with metastasis or migration^40, 41^. However, we could not find any research that used wet-lab experiments to verify the association of *BCL2L11*/*BIM* with metastasis or migration directly. *CDH9* belongs to the cadherin superfamily and the proteins encoded by this gene mediate calcium-dependent cell-cell adhesion, where it is believed that the reduction in cell adhesion correlates with tumor metastasis^42, 43^. However, the role of *CDH9* on tumor invasion and metastasis is unclear, and we could not find any reference that associated *CDH9* with metastasis, migration, or invasion.

**Figure 4:**
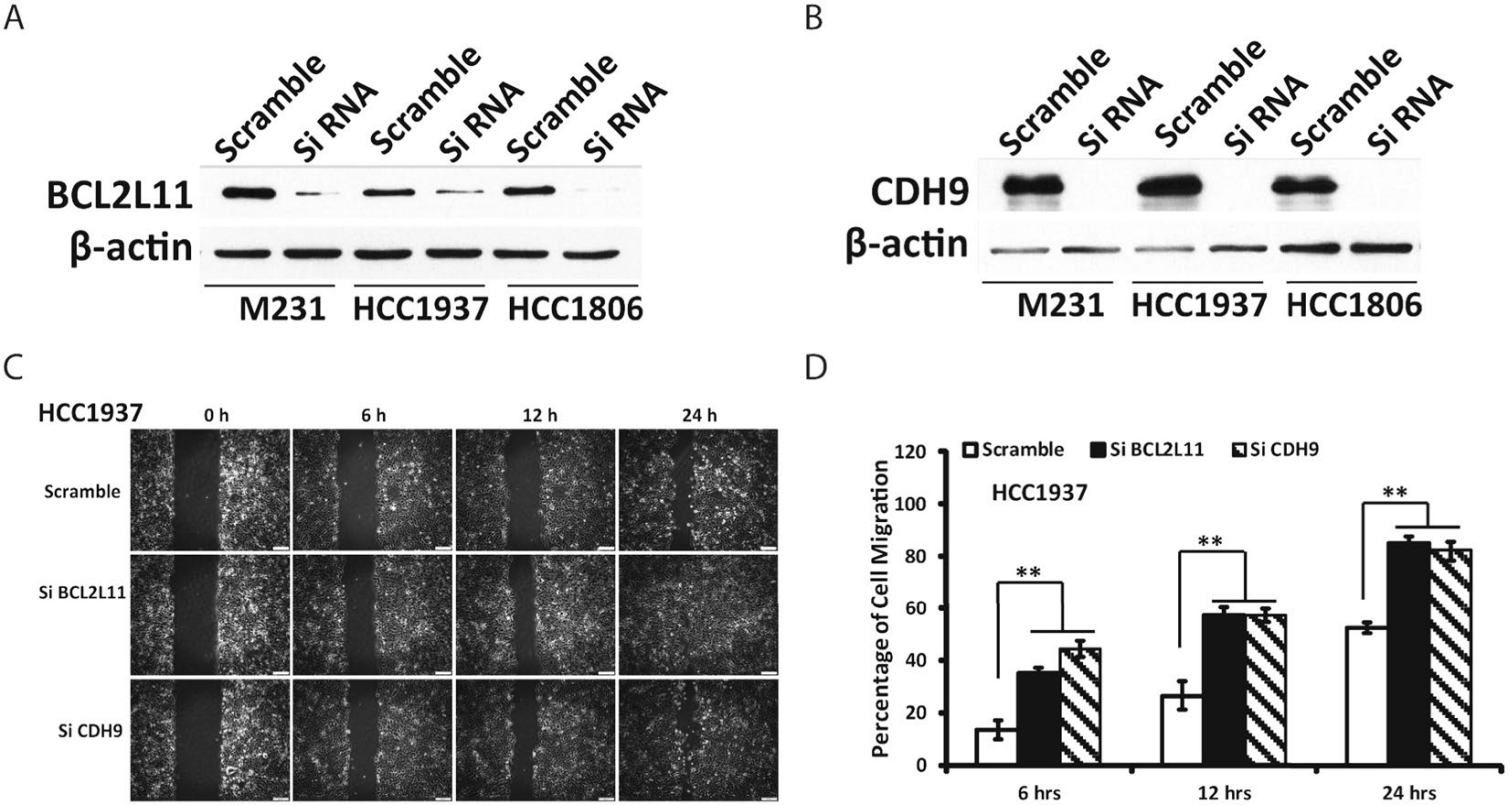
Wound healing assays of HCC1937 cell line with si*BCL2L11*, si*CDH9*, and scramble. (**A**) Western blot showing the impact of siRNA treatment on the expression of gene *BCL2L11*. (**B**) Western blot showing the impact of siRNA treatment on the expression of gene *CDH9*. (**C**,**D**) Impact of si*BCL2L11* or si*CDH9* on the cell migration in cell line HCC1937.

In our results, the gene *BCL2L11* is in driver module 9 and is either mutated or deleted among tumors in the corresponding *T*_*abn*_. The gene *CDH9* is in driver module 17 and mainly mutated among tumors. Hence, we suppose that both genes are metastasis suppressors. In this project, we used *in vitro* wound healing assays to test the association of the genes *BCL2L11* and *CDH9* with cell migration. We found that the siRNA of either of these two genes significantly promoted cell migration of the HCC1937, HCC1806, and MDA-MB-231 cell lines (Fig. 4 and Fig. S2). Hence, both *BCL2L11* and *CDH9* are highly likely to be suppressors of cancer metastasis, which agrees with our hypothesis. To the best of our knowledge, we believe that we are the first to verify that gene *BCL2L11* is a suppressor of cancer metastasis. We are also the first to report and verify that *CDH9* is a suppressor of cancer metastasis.

## Discussion

Many methods have been developed for finding cancer drivers, with each having its advantages and limitations. The frequency-based methods, which search for genes with higher frequency of mutations than those that may be found by chance, are simple to use and easily recognize important drivers with very high SGA frequency^44^. People have developed various ways to improve estimation of the background mutation rate, which is key to avoid false negatives and prevent false positives^45, 46^. However, current frequency-based methods are not intended to identify if drivers are on the same pathway.

Mutual exclusivity is a well-known property that is currently widely used to search for drivers^47–52^. However, even if SGA events perturbing the same pathway have a tendency to be mutually exclusive, this does not mean that SGA events that are mutually exclusive must perturb the same pathway. Furthermore, when the tumor size is large, using only mutual exclusivity to search for drivers can easily group mutually exclusive genes that come from different pathways together. One improvement is combining gene expression information, based on the hypothesis that genes in the same pathway should be co-expressed to a certain degree, while using mutual exclusivity to search for the pathways^48^. Very recently, Kim *et al*. provided a new algorithm to estimate statistical significance of mutual exclusivity relationships^52^. Their new method could find some new drivers, such as TTN (muscle protein Titin), a very long gene that was highly mutated but generally assumed to be artifacts. We also developed an improved method that took into account signal information^51^. However, as this method still uses mutual exclusivity as the major criteria and does not allow any overlap, it misses some important drivers that might have few overlaps with other important drivers.

In this paper, our new method sets the signal information as the major basis in searching for driver modules, but at the same time, we also need the solutions to have a certain degree of mutual exclusivity. In addition to the constraint on the co-expression of genes in each transcriptomic module, we also need those genes to be regulated by a common transcription factor, which increases the likelihood that genes in each transcriptomic module are regulated by a common signal. Hence, each driver module in our results should have very strong information with respect to a common signal. We supposed that genes in a driver module carry a common signal regulating the expression of genes in its corresponding transcriptomic module, and the SGA events of genes in the driver module will perturb the common signal and cause expression changes of genes in the transcriptomic module. We believe that it is the SGA events of genes in the driver modules that cause the expression changes of genes in the transcriptomic modules and not vice versa because SGA events are more stable when compared to the gene expression levels within cells. However, using the TCGA expression and mutation data, any computational method can only identify associations between the SGA events of genes in the driver and the expression changes of genes in the transcriptomic module. Real causal relations need to be verified by experiments. We did not perform these kinds of assays in this work. However, we did verify that the siRNA of two genes in our driver modules have a direct impact on metastatic activities, which proved the feasibility of our new framework.

## Methods

**Figure 5:**
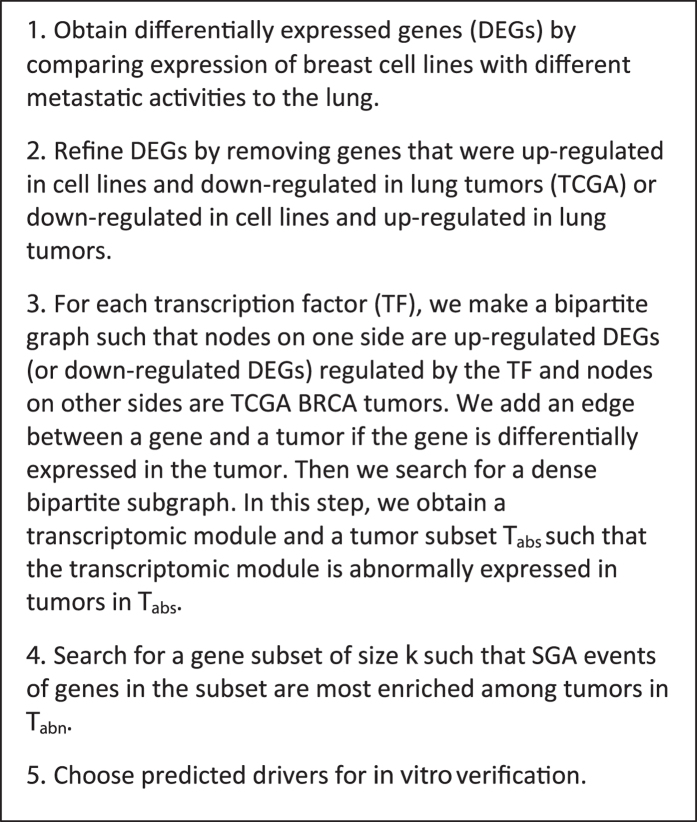
The overall scheme of the framework.

The expression data of cell lines MDA-MB-231 and its subpopulations were obtained from Supplementary Data S1 in Minn’s paper^2^. Data on somatic mutation, copy number alteration, and gene expression from BRCA tumors and normal samples (Note: no mutation and copy number alteration data for normal samples), and data on gene expression from LUSC and LUAD tumors and the corresponding normal controls were downloaded from TCGA^53–55^. The transcription factor (TF) and target genes data were obtained from Osmanbeyoglu’s paper^56^, which was originally obtained from MSigDB^57^ by removing motifs that have similar sets of targets. The overall scheme of the method is shown in Fig. 5.

### Search for gene transcriptomic modules

The parental MDA-MB-231 cell line and its subpopulations were generated through a series of selections *in vivo*, where the parental MDA-MB-231 cell line induced LM0 subpopulations; LM0 subpopulations induced LM1 subpopulations; and LM1 subpopulations induced LM2 subpopulations^2^. Each new generation had more lung metastatic activity than its previous generation. We wanted to catch expression changes of genes in each generation of subpopulations by comparing the expression data of a subpopulation with its direct parental cell lines, such as 1834 cells (LM0) vs. the parental MDA-MB-231 cells or 3481 cells (LM1) vs. 1834 cells (LM0). We considered a gene to be differentially expressed if its expression changed at least 2-fold. We made a union of all up-regulated genes and did the same to the down-regulated gene. Then we removed genes that were both up- and down-regulated in different cases. We obtained a total of 1136 up-regulated and 1154 down-regulated genes. These were the initial candidates for finding gene transcriptomic modules. We set a relatively relaxed threshold in this step to avoid excluding genes that were actually significantly expressed but might be excluded due to error. We use large population tumor data to refine the members in the gene transcriptomic modules in later steps.

We believed that if a gene that is related to breast metastasis to the lung is up-regulated in highly metastatic cell lines, then the gene should not be down-regulated in lung tumors. It is similar for genes that were down-regulated in metastatic cell lines. Therefore, we further removed genes from the initial candidate list if they were up-regulated in cell lines and down-regulated in R% of LUSC or R% of LUAD tumors. We also removed genes from the list if they were down-regulated in cell lines and up-regulated in R% of LUSC or R% of LUAD tumors (we chose R to be 20 in our application, people can adjust this number according to their data and requirement). To decide if a gene was up- or down-regulated in an LUSC tumor, we first found the distribution of the gene in normal controls for LUSC tumors, i.e. the mean *μ* and standard deviation δ of the expression values in normal controls. Then we used the *μ*, δ, and a *p*-value of 0.05 as threshold to decide if a gene was up-regulated or down-regulated in LUSC tumors. We used the same process to identify the up-/down-regulated genes in the LUAD and BRCA tumors. After this step, we had 740 up-regulated genes and 390 down-regulated genes.

We then searched for gene transcriptomic modules where we required that genes in each module were regulated by a common transcription factor (TF) and co-expressed in a large number of breast tumors. Hence, genes in each transcriptomic module are highly likely to be regulated by a common signal. For the up-regulated gene candidates and each TF, we made a bipartite graph such that nodes on one side were up-regulated gene candidates regulated by the TF and nodes on the other side were TCGA BRCA tumors. We added an edge between a gene and a tumor if the gene was differentially expressed in the tumor. Then we searched for a dense bipartite subgraph such that it has at least *n* genes and at least *m* tumors. Furthermore, each gene in the subgraph must be connected to at least *r* × *m* tumors in the subgraph and each tumor in the subgraph must connected to at least *r* × *n* genes in the subgraph. For the dense bipartite subgraph, we obtained a gene subset, which we called the gene transcriptomic module, and a tumor subset, which we denoted as *T*_*abn*_. We also generated another tumor subset called *T*_*nor*_, such that each tumor in *T*_*nor*_ has at most *t* × *n* genes in the gene transcriptomic module that are up-regulated. Our objectives in this step were that: 1) genes in the gene transcriptomic module are regulated by a common signal; 2) tumors in *T*_*abn*_ have SGAs to perturb this common signal; 3) tumors in *T*_*nor*_ do not have SGAs to perturb this common signal. In this work, we set *m*, *n*, *r*, and *t* to be 50, 8, 0.75, and 0.5 respectively, these parameters can be adjusted according to need. We used a similar process for the down-regulated gene candidates.

### Select candidates for driver modules

Before selecting candidates for driver modules, we did a preprocessing of SGA data. The purpose of preprocessing was to exclude invalid SGA events, i.e. SGA events that did not affect the functions of their gene products, proteins, or the expression levels of their corresponding genes. We first excluded all silent mutations. We then removed copy number amplifications/deletions that did not have significant expression increase/decrease for their corresponding genes.

We assumed that tumors in *T*_*abn*_ have SGAs to perturb the common signal regulating a transcriptomic module while tumors in the corresponding *T*_*nor*_ do not. Therefore, if a gene *g* is on the pathway carrying the common signal, then SGA events of gene *g* in BRCA tumors should occur mostly in tumors in *T*_*abn*_ and only in very few tumors that are mistakenly put into *T*_*nor*_ because of a computational error. Enrichment analysis, then, is a reasonable tool to use to measure the information between SGAs of a gene and the common signal regulating the transcriptomic module. We used hypergeometric distribution and *T*_*nor*_ as control background to evaluate the SGA enrichment of each gene among tumors in *T*_*abn*_. We excluded any gene with an SGA enrichment *p*-value larger than 0.01. We chose a strict *p*-value threshold as we wanted to increase the likelihood that the included gene candidates are on the pathway carrying the common signal. Another reason is that we only searched for driver modules with small sizes, such as 6, or 7. A small candidate pool is sufficient and can also improve our final solution.

### Search for driver modules

A reasonable hypothesis is that if a set of genes carries a common signal regulating a transcriptomic module, then the SGA events of all genes in this set should also be enriched among tumors in the corresponding *T*_*abn*_. Furthermore, SGA events of genes on the same pathway usually show mutual exclusivity, i.e. each tumor usually has at most one SGA event to perturb a common signal^47–51^. We developed a genetic algorithm to search for driver modules such that the SGA events of genes in each driver module are highly enriched and mutually exclusive among tumors in a corresponding *T*_*abn*_. We first used a greedy algorithm to generate 2 *S*/3 solutions of size *k* (sets of *k* SGA genes) and a random process to generate another *S*/3 solutions of size *k* (the first generation of chromosomes for the genetic algorithm). The greedy algorithm repeatedly chose genes with the current best weight and without the conflict of mutual exclusivity, where any genes that had been used before were removed from re-selection of new solutions with a probability of 0.5. To create the next generation of solutions, we emulated every pair of current solutions. First we randomly chose a position to make the crossover, which obtained two new solutions. Then, we randomly replaced two genes for one new solution and one gene for the other new solution. Finally, we chose the top *S* solutions from the current solutions and new solutions to make the new generation solutions. For the weight function of the solution, if the size of a solution was less than *k* or the mutually exclusive ratio of the solution was larger than the threshold *r*_*me*_, we set the weight of the solution to be 1; otherwise, we set the weight to be the SGA event enrichment *p*-value of the solution. The mutually exclusive ratio was defined as the ratio of number of tumors in *T*_*abn*_ that are covered by the SGA events of genes in the solution to the total SGA event count of genes in the solution among tumors in *T*_*abn*_. We tested several settings and presented solutions with the setting of *S* = 20, *k* = 6, *r*_*me*_ = 0.8. We ran the algorithm for 6,000 generations.

### Cell migration test for new drivers

#### Cell lines and cell culture

HCC1937, MDA-MB-231 and HCC1806 SPORE cell lines were from the American Type Culture Collection (Manassas, VA, USA). MDA-MB-231 was cultured in DMEM supplemented with 10% fetal bovine serum (FBS). HCC1937 and HCC1806 were maintained in RPMI 1640 supplemented with 10% FBS. The cells were cultured at 37 °C in a humidified atmosphere containing 5% CO_2_.

#### Antibodies

The specific antibody against *BCL2L11* and the secondary antibodies of horseradish peroxidase–conjugated goat anti-mouse and anti-rabbit were from Cell Signaling (Beverly, MA). The *CDH9* antibody was from Thermo Fisher Scientific. The β-actin antibody was purchased from Sigma-Aldrich (St. Louis, MO).

### SiRNA transfection

Smartpool: on-targetplus *BCL2L11*, *CDH9* and control siRNA were purchased from Dharmacon.

Human *BCL2L11* siRNA - SMARTpool, L-004383-00-0005

Human *CDH9* siRNA - SMARTpool, L-013169-00-0005

Non-targeting Pool, D-001810-10-05

SiRNAs were transfected into HCC1937, MDA-MB-231, and HCC1806 cells using Lipofectamine RNAiMAX Reagent (Life technologies).

#### Immunoblotting

The HCC1937, MDA-MB-231 and HCC1806 cells were washed with PBS and collected in a boiling sample buffer 3 days after siRNA transfection. Cellular proteins were resolved by SDS–PAGE (12% acrylamide) and transferred to PVDF membranes (Merck Millipore Ltd). After blocking with 5% non-fat milk in PBST (PBS and 0.1% Tween 20), the membranes were incubated overnight in a cold room with the primary antibodies and for 1 h with the horseradish peroxidase–conjugated secondary antibody. Bound antibodies were detected using Clarity Western ECL substrate (Bio-Rad).

#### Migration assays

A wound healing assay was used to analyze the cell migration of transfectant cells. 4 × 10^5^ of cells were seeded in 35 mm dishes. Cells were transfected with siRNAs 24 h later and cultured for 2 days to a confluence of 90%. The cells were then starved with 0.1% FBS overnight and scratched with a sterile 200-μl micropipette tip to form a straight wound. The cells were washed three times with PBS and cultured in normal medium for an additional 24 h. An Olympus IX83 microscope was used to measure the wound closure. Images were recorded at the time points of 0, 6, 12 and 24 h after wounding. The distances invaded by the cells at the front of the wound were measured from the control and the experimental samples. Cell migration was assayed by calculating the migrated distance and comparing with time 0.

#### Statistic test

The differences between the control and treated groups was evaluated using a *t*-test. Statistical significance was calculated based on three experiments. A *p-*value of < 0.05 was considered statistically significant.

## Electronic supplementary material

Supplementary
