# Supplementary material for: A signal-based method for finding driver modules of breast cancer metastasis to the lung
Source: Sci Rep. 2017 Aug 30;7:10023. doi: 10.1038/s41598-017-09951-2 (PMC5577160; doi:10.1038/s41598-017-09951-2)
Supplement: Supplementary file 1 — Supplementary [file 41598_2017_9951_MOESM1_ESM.pdf]

A signal-based method for finding driver modules of breast cancer metastasis to the lung

Gaibo Yan[1,2], Vicky Chen[1,2], Xinghua Lu[1,2], Songjian Lu[1,2,*]

[1]Department of Biomedical Informatics, University of Pittsburgh, [2]Center for Causal Discovery, University of Pittsburgh. Pittsburgh, PA, USA.

[*]Corresponding Authors: Songjian Lu

Email: songjian@pitt.edu

Running title: Find drivers of breast cancer metastasis to lung

Keywords: cancer metastasis, transcriptomic module, common signal, driver genome alterations.

Supplementary

Cell migration test for new drivers

Cell lines and cell culture: HCC1937, MDA-MB-231 and HCC1806 SPORE cell lines were from the American Type Culture Collection (Manassas, VA, USA). The MDA-MB-231 were cultured in DMEM supplemented with 10% fetal bovine serum (FBS). HCC1937 and HCC1806 were maintained in RPMI 1640 supplemented with 10% FBS. The cells were cultured at 37°C in a humidified atmosphere containing 5% CO_2.

Antibodies: The specific antibody against *BCL2L11* and the secondary antibodies of Horseradish peroxidase–conjugated goat anti-mouse and anti-rabbit were from Cell Signaling (Beverly, MA). The *CDH9* antibody was from Thermo Fisher Scientific. The β-actin antibody was purchased from Sigma-Aldrich (St. Louis, MO).

SiRNA transfection:

Smartpool: on-targetplus *BCL2L11*, *CDH9* and control siRNA were purchased from Dharmacon.

Human *BCL2L11* siRNA - SMARTpool, L-004383-00-0005

Human *CDH9* siRNA - SMARTpool, L-013169-00-0005

Non-targeting Pool, D-001810-10-05

SiRNAs were transfected into HCC1937, MDA-MB-231, and HCC1806 cells using Lipofectamine RNAiMAX Reagent (Life technologies).

Immunoblotting: The HCC1937, MDA-MB-231 and HCC1806 cells were washed with PBS and collected in a boiling sample buffer 3 days after siRNA transfection. Cellular proteins were resolved by SDS–PAGE (12% acrylamide) and transferred to PVDF membranes (Merck Millipore Ltd). After blocking with 5% non-fat milk in PBST (PBS and 0.1% Tween 20), the membranes were incubated overnight in a cold room with the primary antibodies and for 1 h with the horseradish peroxidase–conjugated secondary antibody. Bound antibodies were detected using Clarity Western ECL substrate (Bio-Rad).

Migration assays: A wound healing assay was used to analyze the cell migration of transfectant cells. $4x10^5$ of cells were seeded in 35 mm dishes. Cells were transfected with siRNAs 24 hrs later and cultured for 2 days to a confluence of 90%. The cells were then starved with 0.1% FBS overnight and scratched with a sterile 200-μl micropipette tip to form a straight wound. The cells were washed three times with PBS and cultured in normal medium for an additional 24 h. An Olympus IX83 microscope was used to measure the wound closure. Images were recorded at the time points of 0, 6, 12 and 24 h after wounding. The distances invaded by the cells at the front of the wound were measured from the control and the experimental samples. Cell migration was assayed by calculating the migrated distance and comparing with time 0.

Statistic test: The differences between the control and treated groups was evaluated using a *t*-test. Statistical significance was calculated based on three experiments. A *p*-value of <0.05 was considered statistically significant.

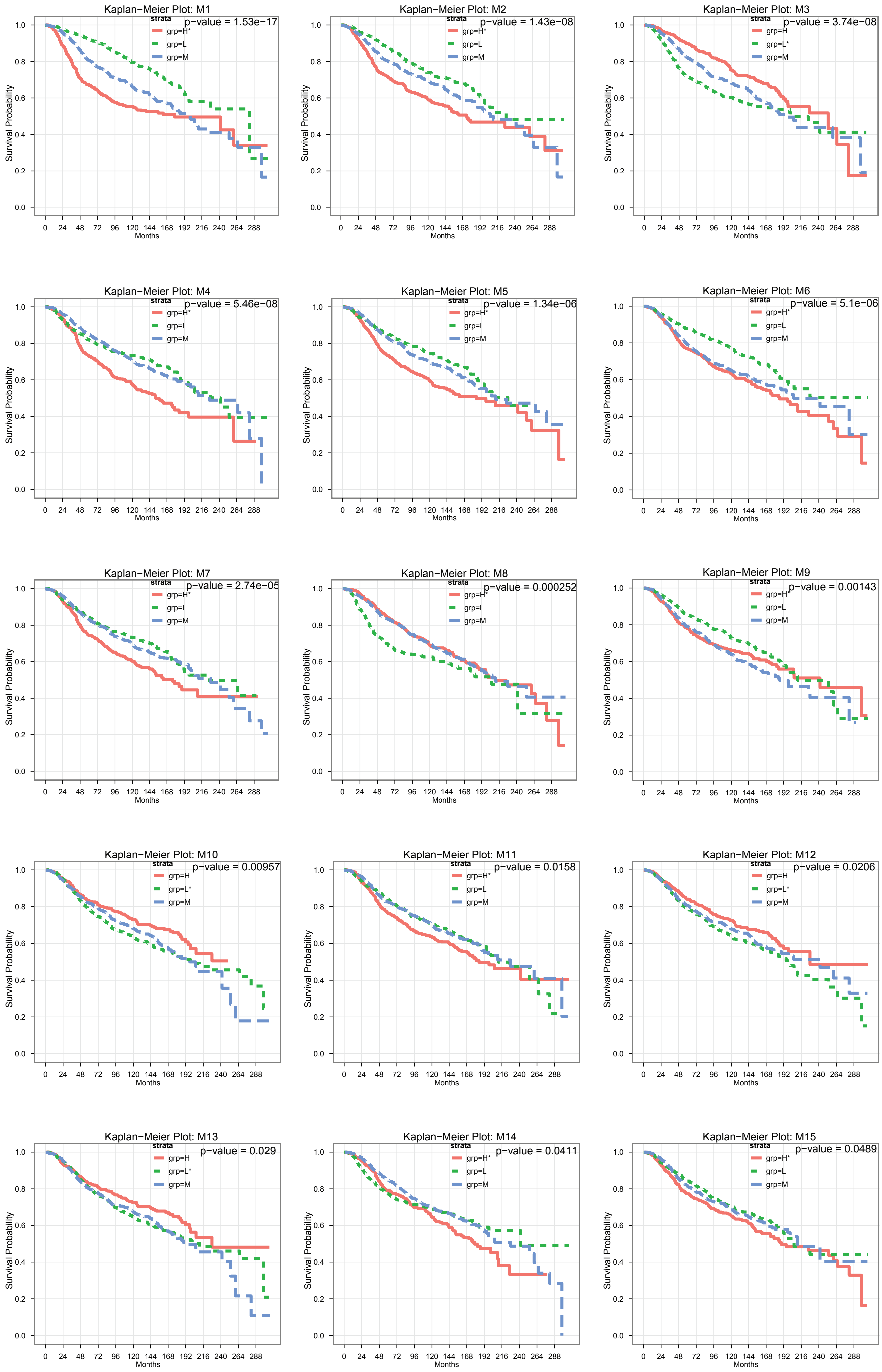
Kaplan−Meier Plot: M1
p−value = 1.53e−17
strata
grp=H*
grp=L
grp=M
Kaplan−Meier Plot: M2
p−value = 1.43e−08
strata
grp=H*
grp=L
grp=M
Kaplan−Meier Plot: M3
p−value = 3.74e−08
strata
grp=H
grp=L*
grp=M
Kaplan−Meier Plot: M4
p−value = 5.46e−08
strata
grp=H*
grp=L
grp=M
Kaplan−Meier Plot: M5
p−value = 1.34e−06
strata
grp=H*
grp=L
grp=M
Kaplan−Meier Plot: M6
p−value = 5.1e−06
strata
grp=H*
grp=L
grp=M
Kaplan−Meier Plot: M7
p−value = 2.74e−05
strata
grp=H*
grp=L
grp=M
Kaplan−Meier Plot: M8
p−value = 0.000252
strata
grp=H*
grp=L
grp=M
Kaplan−Meier Plot: M9
p−value = 0.00143
strata
grp=H*
grp=L
grp=M
Kaplan−Meier Plot: M10
p−value = 0.00957
strata
grp=H
grp=L*
grp=M
Kaplan−Meier Plot: M11
p−value = 0.0158
strata
grp=H*
grp=L
grp=M
Kaplan−Meier Plot: M12
p−value = 0.0206
strata
grp=H
grp=L*
grp=M
Kaplan−Meier Plot: M13
p−value = 0.029
strata
grp=H
grp=L*
grp=M
Kaplan−Meier Plot: M14
p−value = 0.0411
strata
grp=H*
grp=L
grp=M
Kaplan−Meier Plot: M15
p−value = 0.0489
strata
grp=H*
grp=L
grp=M
Survival Probability
0.0 0.2 0.4 0.6 0.8 1.0
Months
0 24 48 72 96 120 144 168 192 216 240 264 288

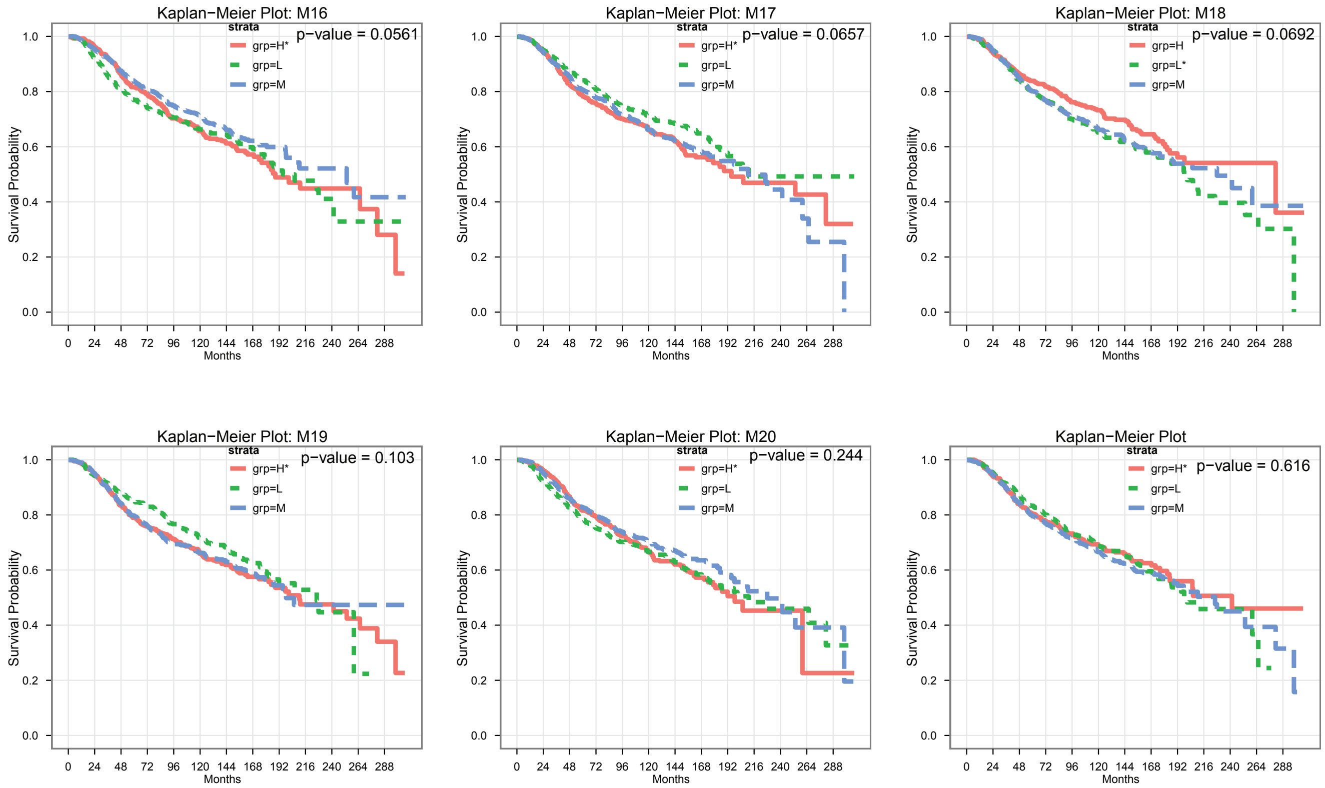

Fig. S1: **The impact of expression statuses of gene transcriptomic modules to the patients' survival**. Patient groups with high, middle, low expressions of a transcriptomic module are represented as H, M, L, respectively. H* means that the module is up-regulated in cell lines with high metastatic activities. L* means that the module is down-regulated in cell lines with high metastatic activities.

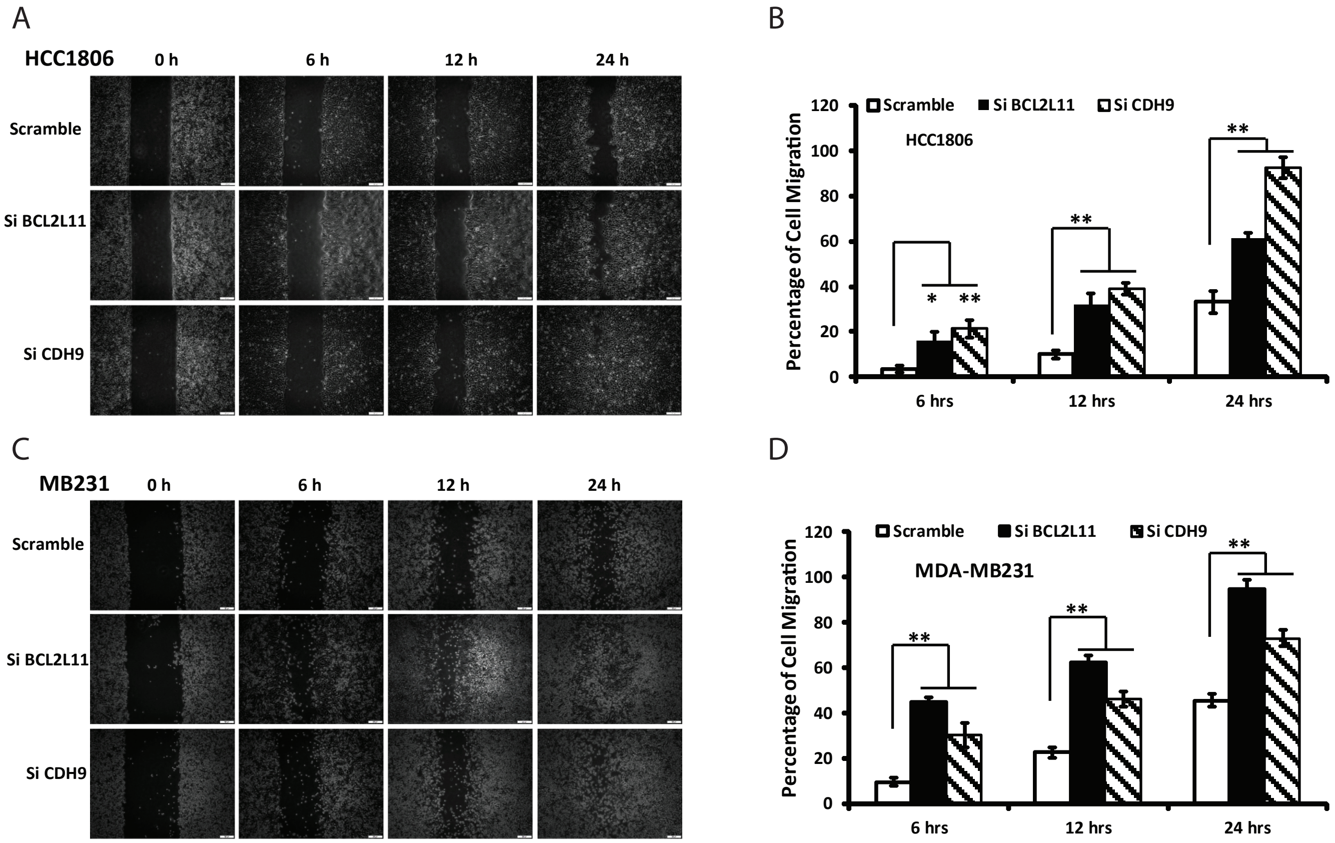

Fig. S2: Wound healing assays of HCC1806 and MDA-MB-231 cell lines. A B) siBCL2L11, siCDH9 of HCC1806 cell line promote EMT significantly. C D) siBCL2L11, siCDH9 of MDA-MB-231 cell line promote EMT significantly.

Table S1: Literature search reveals that many genes in our driver modules are related to metastasis

Modu ID	References	Count
1	LSR[1-3], PTP4A3[4-7], S100A14[8-10], SMCP[11], TP53[12-16], ZWINT	5
2	ABCF3, ATG7[17,18], ERBB2[19-22], NME1[23-26], ODF2[27,28], VPS72	4
3	FAM49B, NME1[23-26], STARD7[29], TMEM189, ZFYVE16, ZNF596	2
4	C20orf201, ERBB2[19-22], FBN1, HOOK1[30], RP9, ZBTB7B	2
5	COIL, GRB7[31-34], IL20[35-37], PYCR2, SH2B1[38], TPCN2	3
6	CGN, GNA13[39-41], GRHL2[42-44], ITM2B, MTL5, TNFRSF10B[45]	3
7	EPPK1, ERBB2[19-22], IMPG2, KIAA0195, POLDIP2[46,47], PYGB	2
8	ANO1[48-50], ATAD5, CLCF1, IARS, PIK3CA[51-55], TRIM58	2
9	BCL2L11[56-58], DISP2, IL2RA[59-62], PLEKHG6[63-65], SMC1B, TMEM105	3
10	APOA1BP, MRPL55, RBM4B, SHARPIN[66,67], SMARCD2, XPO7[68]	2
11	CCNI, DPT[69,70], LIMD2, LRCH4, LY96[71,72], RUSC2	2
12	GNRHR2, LRRC42, PPP1R3B, SDHAP1, STMN3[73,74], TRPS1[75-77]	2
13	ARHGEF10, CBWD1, EIF4A3, RECQL4[78], RUSC1, TRPS1[75-77]	2
14	ATP1B4, ERLIN2, IFT88, RNFT1, SLC7A13, VIL1[79]	1
15	DOCK11, GRB7[31-34], MAP3K13, SLC39A11, TAB2[80,81], ZCCHC11[82,83]	3
16	C8orf86, CACNG4, CHAF1A[84], FAM5B, SMYD3[85-87], ZNF835	2
17	CAPN8, CDH9, COQ10B, HOXB3[88-90], IPPK, SGK494	1
18	BAIAP2[91], EEF1A2[92-94], GUK1[95], PPP1R3B, TEX2, TRPS1[75-77]	4
19	ADCK5, CHMP6, NDUFS6, RAB13[96-98], TDRD12, TRMT1	1
20	PIK3CA[51-55], RGS16[99-101], STARD3[102,103], TANC2, TGFB2[104-106], TRIM58	4
21	C1S[107-109], LY96[71,72], PLP2[110-112], PPP3CA[113], SLAMF8, ZBP1[114-117]	5

References

1. Bayat B, Werth S, Sachs UJ, Newman DK, Newman PJ, Santoso S. Neutrophil transmigration mediated by the neutrophil-specific antigen CD177 is influenced by the endothelial S536N dimorphism of platelet endothelial cell adhesion molecule-1. *Journal of immunology.* Apr 1 2010;184(7):3889-3896.
2. Garcia JM, Pena C, Garcia V, et al. Prognostic value of LISCH7 mRNA in plasma and tumor of colon cancer patients. *Clinical cancer research : an official journal of the American Association for Cancer Research.* Nov 1 2007;13(21):6351-6358.
3. Herbsleb M, Birkenkamp-Demtroder K, Thykjaer T, et al. Increased cell motility and invasion upon knockdown of lipolysis stimulated lipoprotein receptor (LSR) in SW780 bladder cancer cells. *BMC medical genomics.* 2008;1:31.
4. Guzinska-Ustymowicz K, Pryczynicz A, Kemona A. PTP4A3 expression increases strongly in lymph node metastases from colorectal carcinoma. *Anticancer research.* Oct 2009;29(10):3913-3916.
5. Cramer JM, Zimmerman MW, Thompson T, Homanics GE, Lazo JS, Lagasse E. Deletion of Ptp4a3 reduces clonogenicity and tumor-initiation ability of colitis-associated cancer cells in mice. *Stem cell research.* Jul 2014;13(1):164-171.
6. Laurent C, Valet F, Planque N, et al. High PTP4A3 phosphatase expression correlates with metastatic risk in uveal melanoma patients. *Cancer Res.* Feb 1 2011;71(3):666-674.
7. Zimmerman MW, Homanics GE, Lazo JS. Targeted deletion of the metastasis-associated phosphatase Ptp4a3 (PRL-3) suppresses murine colon cancer. *PloS one.* 2013;8(3):e58300.
8. Wang HY, Zhang JY, Cui JT, et al. Expression status of S100A14 and S100A4 correlates with metastatic potential and clinical outcome in colorectal cancer after surgery. *Oncology reports.* Jan 2010;23(1):45-52.
9. Tanaka M, Ichikawa-Tomikawa N, Shishito N, et al. Co-expression of S100A14 and S100A16 correlates with a poor prognosis in human breast cancer and promotes cancer cell invasion. *BMC cancer.* 2015;15:53.
10. Xu C, Chen H, Wang X, et al. S100A14, a member of the EF-hand calcium-binding proteins, is overexpressed in breast cancer and acts as a modulator of HER2 signaling. *The Journal of biological chemistry.* Jan 10 2014;289(2):827-837.
11. Takahashi A, Hirohashi Y, Torigoe T, et al. Ectopically expressed variant form of sperm mitochondria-associated cysteine-rich protein augments tumorigenicity of the stem cell population of lung adenocarcinoma cells. *PLoS One.* 2013;8(11):e69095.
12. Muller PA, Vousden KH. p53 mutations in cancer. *Nature cell biology.* Jan 2013;15(1):2-8.
13. Wang SP, Wang WL, Chang YL, et al. p53 controls cancer cell invasion by inducing the MDM2-mediated degradation of Slug. *Nature cell biology.* Jun 2009;11(6):694-704.
14. Mizuarai S, Yamanaka K, Kotani H. Mutant p53 induces the GEF-H1 oncogene, a guanine nucleotide exchange factor-H1 for RhoA, resulting in accelerated cell proliferation in tumor cells. *Cancer research.* Jun 15 2006;66(12):6319-6326.
15. Grawenda AM, Moller EK, Lam S, et al. Interaction between p53 mutation and a somatic HDMX biomarker better defines metastatic potential in breast cancer. *Cancer research.* Feb 15 2015;75(4):698-708.
16. Arjonen A, Kaukonen R, Mattila E, et al. Mutant p53-associated myosin-X upregulation promotes breast cancer invasion and metastasis. *The Journal of clinical investigation.* Mar 2014;124(3):1069-1082.

17. Tuloup-Minguez V, Hamai A, Greffard A, Nicolas V, Codogno P, Botti J. Autophagy modulates cell migration and beta1 integrin membrane recycling. *Cell cycle.* Oct 15 2013;12(20):3317-3328.
18. Palumbo S, Tini P, Toscano M, et al. Combined EGFR and autophagy modulation impairs cell migration and enhances radiosensitivity in human glioblastoma cells. *Journal of cellular physiology.* Nov 2014;229(11):1863-1873.
19. De Greve J, Teugels E, Geers C, et al. Clinical activity of afatinib (BIBW 2992) in patients with lung adenocarcinoma with mutations in the kinase domain of HER2/neu. *Lung cancer.* Apr 2012;76(1):123-127.
20. Schade B, Lesurf R, Sanguin-Gendreau V, et al. beta-Catenin signaling is a critical event in ErbB2-mediated mammary tumor progression. *Cancer research.* Jul 15 2013;73(14):4474-4487.
21. Gillibert-Duplantier J, Duthey B, Sisirak V, et al. Gene expression profiling identifies sST2 as an effector of ErbB2-driven breast carcinoma cell motility, associated with metastasis. *Oncogene.* Jul 26 2012;31(30):3516-3524.
22. Nelson SE, Gould MN, Hampton JM, Trentham-Dietz A. A case-control study of the HER2 Ile655Val polymorphism in relation to risk of invasive breast cancer. *Breast cancer research : BCR.* 2005;7(3):R357-364.
23. Chandrasekharappa SC, Gross LA, King SE, Collins FS. The human NME2 gene lies within 18kb of NME1 in chromosome 17. *Genes, chromosomes & cancer.* Apr 1993;6(4):245-248.
24. Rosengard AM, Krutzsch HC, Shearn A, et al. Reduced Nm23/Awd protein in tumour metastasis and aberrant Drosophila development. *Nature.* Nov 9 1989;342(6246):177-180.
25. Kelsell DP, Black DM, Solomon E, Spurr NK. Localization of a second NM23 gene, NME2, to chromosome 17q21-q22. *Genomics.* Aug 1993;17(2):522-524.
26. Backer JM, Mendola CE, Kovesdi I, et al. Chromosomal localization and nucleoside diphosphate kinase activity of human metastasis-suppressor genes NM23-1 and NM23-2. *Oncogene.* Feb 1993;8(2):497-502.
27. Smith TC, Fang Z, Luna EJ. Novel interactors and a role for supervillin in early cytokinesis. *Cytoskeleton.* Jun 2010;67(6):346-364.
28. Ghafouri-Fard S, Abbasi A, Moslehi H, et al. Elevated expression levels of testis-specific genes TEX101 and SPATA19 in basal cell carcinoma and their correlation with clinical and pathological features. *The British journal of dermatology.* Apr 2010;162(4):772-779.
29. Flores-Martin J, Rena V, Marquez S, Panzetta-Dutari GM, Genti-Raimondi S. StarD7 knockdown modulates ABCG2 expression, cell migration, proliferation, and differentiation of human choriocarcinoma JEG-3 cells. *PloS one.* 2012;7(8):e44152.
30. Zhou Y, Hu Y, Yang M, et al. The miR-106b~25 cluster promotes bypass of doxorubicin-induced senescence and increase in motility and invasion by targeting the E-cadherin transcriptional activator EP300. *Cell death and differentiation.* Mar 2014;21(3):462-474.
31. Pias S, Peterson TA, Johnson DL, Lyons BA. The intertwining of structure and function: proposed helix-swapping of the SH2 domain of Grb7, a regulatory protein implicated in cancer progression and inflammation. *Critical reviews in immunology.* 2010;30(3):299-304.
32. Zhang D, Shao C, Hu S, Ma S, Gao Y. Novel nonphosphorylated peptides with conserved sequences selectively bind to Grb7 SH2 domain with affinity comparable to its phosphorylated ligand. *PloS one.* 2012;7(1):e29902.
33. Tanaka S, Mori M, Akiyoshi T, et al. Coexpression of Grb7 with epidermal growth factor receptor or Her2/erbB2 in human advanced esophageal carcinoma. *Cancer research.* Jan 1 1997;57(1):28-31.
34. Tanaka S, Mori M, Akiyoshi T, et al. A novel variant of human Grb7 is associated with invasive esophageal carcinoma. *The Journal of clinical investigation.* Aug 15 1998;102(4):821-827.

35. Hsieh MY, Chen WY, Jiang MJ, Cheng BC, Huang TY, Chang MS. Interleukin-20 promotes angiogenesis in a direct and indirect manner. *Genes and immunity.* Apr 2006;7(3):234-242.
36. Lee SJ, Cho SC, Lee EJ, et al. Interleukin-20 promotes migration of bladder cancer cells through extracellular signal-regulated kinase (ERK)-mediated MMP-9 protein expression leading to nuclear factor (NF-kappaB) activation by inducing the up-regulation of p21(WAF1) protein expression. *The Journal of biological chemistry.* Feb 22 2013;288(8):5539-5552.
37. Lee SJ, Lee EJ, Kim SK, et al. Identification of pro-inflammatory cytokines associated with muscle invasive bladder cancer; the roles of IL-5, IL-20, and IL-28A. *PloS one.* 2012;7(9):e40267.
38. Zhang H, Duan CJ, Chen W, et al. Clinical significance of SH2B1 adaptor protein expression in non-small cell lung cancer. *Asian Pacific journal of cancer prevention : APJCP.* 2012;13(5):2355-2362.
39. Zhang JX, Mai SJ, Huang XX, et al. MiR-29c mediates epithelial-to-mesenchymal transition in human colorectal carcinoma metastasis via PTP4A and GNA13 regulation of beta-catenin signaling. *Annals of oncology : official journal of the European Society for Medical Oncology / ESMO.* Nov 2014;25(11):2196-2204.
40. Rasheed SA, Teo CR, Beillard EJ, Voorhoeve PM, Casey PJ. MicroRNA-182 and microRNA-200a control G-protein subunit alpha-13 (GNA13) expression and cell invasion synergistically in prostate cancer cells. *The Journal of biological chemistry.* Mar 15 2013;288(11):7986-7995.
41. Rasheed SA, Teo CR, Beillard EJ, et al. MicroRNA-31 controls G protein alpha-13 (GNA13) expression and cell invasion in breast cancer cells. *Molecular cancer.* 2015;14:67.
42. Werner S, Frey S, Riethdorf S, et al. Dual roles of the transcription factor grainyhead-like 2 (GRHL2) in breast cancer. *The Journal of biological chemistry.* Aug 9 2013;288(32):22993-23008.
43. Varma S, Cao Y, Tagne JB, et al. The transcription factors Grainyhead-like 2 and NK2-homeobox 1 form a regulatory loop that coordinates lung epithelial cell morphogenesis and differentiation. *The Journal of biological chemistry.* Oct 26 2012;287(44):37282-37295.
44. Xiang X, Deng Z, Zhuang X, et al. Grhl2 determines the epithelial phenotype of breast cancers and promotes tumor progression. *PloS one.* 2012;7(12):e50781.
45. Tahir RA, Sehgal SA, Khattak NA, Khan Khattak JZ, Mir A. Tumor necrosis factor receptor superfamily 10B (TNFRSF10B): an insight from structure modeling to virtual screening for designing drug against head and neck cancer. *Theoretical biology & medical modelling.* 2013;10:38.
46. Amanso AM, Lassegue B, Joseph G, et al. Polymerase delta-interacting protein 2 promotes postischemic neovascularization of the mouse hindlimb. *Arteriosclerosis, thrombosis, and vascular biology.* Jul 2014;34(7):1548-1555.
47. Lyle AN, Deshpande NN, Taniyama Y, et al. Poldip2, a novel regulator of Nox4 and cytoskeletal integrity in vascular smooth muscle cells. *Circulation research.* Jul 31 2009;105(3):249-259.
48. Shiwarski DJ, Shao C, Bill A, et al. To "grow" or "go": TMEM16A expression as a switch between tumor growth and metastasis in SCCHN. *Clinical cancer research : an official journal of the American Association for Cancer Research.* Sep 1 2014;20(17):4673-4688.
49. Ruiz C, Martins JR, Rudin F, et al. Enhanced expression of ANO1 in head and neck squamous cell carcinoma causes cell migration and correlates with poor prognosis. *PloS one.* 2012;7(8):e43265.
50. Li Y, Zhang J, Hong S. ANO1 as a marker of oral squamous cell carcinoma and silencing ANO1 suppresses migration of human SCC-25 cells. *Medicina oral, patologia oral y cirugia bucal.* Jul 2014;19(4):e313-319.
51. Juric D, Castel P, Griffith M, et al. Convergent loss of PTEN leads to clinical resistance to a PI(3)Kalpha inhibitor. *Nature.* Feb 12 2015;518(7538):240-244.

52. Graupera M, Guillermet-Guibert J, Foukas LC, et al. Angiogenesis selectively requires the p110alpha isoform of PI3K to control endothelial cell migration. *Nature.* May 29 2008;453(7195):662-666.
53. Stevens KN, Garcia-Closas M, Fredericksen Z, et al. Evaluation of variation in the phosphoinositide-3-kinase catalytic subunit alpha oncogene and breast cancer risk. *British journal of cancer.* Dec 6 2011;105(12):1934-1939.
54. Ricarte-Filho JC, Ryder M, Chitale DA, et al. Mutational profile of advanced primary and metastatic radioactive iodine-refractory thyroid cancers reveals distinct pathogenetic roles for BRAF, PIK3CA, and AKT1. *Cancer research.* Jun 1 2009;69(11):4885-4893.
55. Saal LH, Holm K, Maurer M, et al. PIK3CA mutations correlate with hormone receptors, node metastasis, and ERBB2, and are mutually exclusive with PTEN loss in human breast carcinoma. *Cancer research.* Apr 1 2005;65(7):2554-2559.
56. Drury LJ, Wendt MK, Dwinell MB. CXCL12 chemokine expression and secretion regulates colorectal carcinoma cell anoikis through Bim-mediated intrinsic apoptosis. *PloS one.* 2010;5(9):e12895.
57. Woods NT, Yamaguchi H, Lee FY, Bhalla KN, Wang HG. Anoikis, initiated by Mcl-1 degradation and Bim induction, is deregulated during oncogenesis. *Cancer Res.* Nov 15 2007;67(22):10744-10752.
58. Kojima T, Shimazui T, Horie R, et al. FOXO1 and TCF7L2 genes involved in metastasis and poor prognosis in clear cell renal cell carcinoma. *Genes, chromosomes & cancer.* Apr 2010;49(4):379-389.
59. Zhu J, Feng A, Sun J, et al. Increased CD4(+) CD69(+) CD25(-) T cells in patients with hepatocellular carcinoma are associated with tumor progression. *Journal of gastroenterology and hepatology.* Oct 2011;26(10):1519-1526.
60. Powell DJ, Jr., Felipe-Silva A, Merino MJ, et al. Administration of a CD25-directed immunotoxin, LMB-2, to patients with metastatic melanoma induces a selective partial reduction in regulatory T cells in vivo. *Journal of immunology.* Oct 1 2007;179(7):4919-4928.
61. Kadioglu A, Coward W, Colston MJ, Hewitt CR, Andrew PW. CD4-T-lymphocyte interactions with pneumolysin and pneumococci suggest a crucial protective role in the host response to pneumococcal infection. *Infection and immunity.* May 2004;72(5):2689-2697.
62. Kozawa E, Sugiura H, Wasa J, et al. Suppression of tumour metastasis in a murine osteosarcoma model with anti-CD25 monoclonal antibody treatment. *Anticancer research.* Dec 2010;30(12):5019-5022.
63. Zhang P, Ma Y, Wang F, et al. Comprehensive gene and microRNA expression profiling reveals the crucial role of hsa-let-7i and its target genes in colorectal cancer metastasis. *Molecular biology reports.* Feb 2012;39(2):1471-1478.
64. Wu D, Asiedu M, Wei Q. Myosin-interacting guanine exchange factor (MyoGEF) regulates the invasion activity of MDA-MB-231 breast cancer cells through activation of RhoA and RhoC. *Oncogene.* Jun 4 2009;28(22):2219-2230.
65. Wu D, Haruta A, Wei Q. GIPC1 interacts with MyoGEF and promotes MDA-MB-231 breast cancer cell invasion. *The Journal of biological chemistry.* Sep 10 2010;285(37):28643-28650.
66. Pouwels J, De Franceschi N, Rantakari P, et al. SHARPIN regulates uropod detachment in migrating lymphocytes. *Cell reports.* Nov 14 2013;5(3):619-628.
67. De Melo J, Wu V, He L, Yan J, Tang D. SIPL1 enhances the proliferation, attachment, and migration of CHO cells by inhibiting PTEN function. *International journal of molecular medicine.* Sep 2014;34(3):835-841.

68. Hattangadi SM, Martinez-Morilla S, Patterson HC, et al. Histones to the cytosol: exportin 7 is essential for normal terminal erythroid nuclear maturation. *Blood.* Sep 18 2014;124(12):1931-1940.
69. Lieveld M, Bodson E, De Boeck G, et al. Gene expression profiling of giant cell tumor of bone reveals downregulation of extracellular matrix components decorin and lumican associated with lung metastasis. *Virchows Archiv : an international journal of pathology.* Dec 2014;465(6):703-713.
70. Yamatoji M, Kasamatsu A, Kouzu Y, et al. Dermatopontin: a potential predictor for metastasis of human oral cancer. *International journal of cancer. Journal international du cancer.* Jun 15 2012;130(12):2903-2911.
71. Grondin V, Seksik P, Dumont S, et al. Regulation of colon cancer cell proliferation and migration by MD-2 activity. *Innate immunity.* Aug 2011;17(4):414-422.
72. Hsu RY, Chan CH, Spicer JD, et al. LPS-induced TLR4 signaling in human colorectal cancer cells increases beta1 integrin-mediated cell adhesion and liver metastasis. *Cancer research.* Mar 1 2011;71(5):1989-1998.
73. Zhang Y, Ni S, Huang B, et al. Overexpression of SCLIP promotes growth and motility in glioblastoma cells. *Cancer biology & therapy.* 2015;16(1):97-105.
74. Nair S, Bora-Singhal N, Perumal D, Chellappan S. Nicotine-mediated invasion and migration of non-small cell lung carcinoma cells by modulating STMN3 and GSPT1 genes in an ID1-dependent manner. *Molecular cancer.* 2014;13:173.
75. Hu J, Su P, Jia M, et al. TRPS1 expression promotes angiogenesis and affects VEGFA expression in breast cancer. *Experimental biology and medicine.* Apr 2014;239(4):423-429.
76. Hong J, Sun J, Huang T. Increased expression of TRPS1 affects tumor progression and correlates with patients' prognosis of colon cancer. *BioMed research international.* 2013;2013:454085.
77. Bonnard C, Strobl AC, Shboul M, et al. Mutations in IRX5 impair craniofacial development and germ cell migration via SDF1. *Nature genetics.* Jun 2012;44(6):709-713.
78. Su Y, Meador JA, Calaf GM, et al. Human RecQL4 helicase plays critical roles in prostate carcinogenesis. *Cancer research.* Nov 15 2010;70(22):9207-9217.
79. Xieraili M, Yasen M, Mogushi K, et al. Villin 1 is a predictive factor for the recurrence of high serum alpha-fetoprotein-associated hepatocellular carcinoma after hepatectomy. *Cancer science.* Aug 2012;103(8):1493-1501.
80. Morioka S, Inagaki M, Komatsu Y, Mishina Y, Matsumoto K, Ninomiya-Tsuji J. TAK1 kinase signaling regulates embryonic angiogenesis by modulating endothelial cell survival and migration. *Blood.* Nov 1 2012;120(18):3846-3857.
81. Roh YS, Song J, Seki E. TAK1 regulates hepatic cell survival and carcinogenesis. *Journal of gastroenterology.* Feb 2014;49(2):185-194.
82. Piskounova E, Polytarchou C, Thornton JE, et al. Lin28A and Lin28B inhibit let-7 microRNA biogenesis by distinct mechanisms. *Cell.* Nov 23 2011;147(5):1066-1079.
83. Fu X, Meng Z, Liang W, et al. miR-26a enhances miRNA biogenesis by targeting Lin28B and Zcchc11 to suppress tumor growth and metastasis. *Oncogene.* Aug 21 2014;33(34):4296-4306.
84. Wu Z, Cui F, Yu F, et al. Up-regulation of CHAF1A, a poor prognostic factor, facilitates cell proliferation of colon cancer. *Biochemical and biophysical research communications.* Jun 27 2014;449(2):208-215.
85. Cock-Rada AM, Medjkane S, Janski N, et al. SMYD3 promotes cancer invasion by epigenetic upregulation of the metalloproteinase MMP-9. *Cancer research.* Feb 1 2012;72(3):810-820.
86. Luo XG, Zhang CL, Zhao WW, et al. Histone methyltransferase SMYD3 promotes MRTF-A-mediated transactivation of MYL9 and migration of MCF-7 breast cancer cells. *Cancer letters.* Mar 1 2014;344(1):129-137.

87. Zou JN, Wang SZ, Yang JS, Luo XG, Xie JH, Xi T. Knockdown of SMYD3 by RNA interference down-regulates c-Met expression and inhibits cells migration and invasion induced by HGF. *Cancer letters.* Jul 18 2009;280(1):78-85.
88. Weiss FU, Marques IJ, Woltering JM, et al. Retinoic acid receptor antagonists inhibit miR-10a expression and block metastatic behavior of pancreatic cancer. *Gastroenterology.* Dec 2009;137(6):2136-2145 e2131-2137.
89. Chen J, Zhu S, Jiang N, Shang Z, Quan C, Niu Y. HoxB3 promotes prostate cancer cell progression by transactivating CDCA3. *Cancer letters.* Apr 28 2013;330(2):217-224.
90. Manley NR, Capecchi MR. Hox group 3 paralogs regulate the development and migration of the thymus, thyroid, and parathyroid glands. *Developmental biology.* Mar 1 1998;195(1):1-15.
91. Funato Y, Terabayashi T, Suenaga N, Seiki M, Takenawa T, Miki H. IRSp53/Eps8 complex is important for positive regulation of Rac and cancer cell motility/invasiveness. *Cancer Res.* Aug 1 2004;64(15):5237-5244.
92. Xu C, Hu DM, Zhu Q. eEF1A2 promotes cell migration, invasion and metastasis in pancreatic cancer by upregulating MMP-9 expression through Akt activation. *Clinical & experimental metastasis.* Oct 2013;30(7):933-944.
93. Amiri A, Noei F, Jeganathan S, Kulkarni G, Pinke DE, Lee JM. eEF1A2 activates Akt and stimulates Akt-dependent actin remodeling, invasion and migration. *Oncogene.* May 10 2007;26(21):3027-3040.
94. Kawamura M, Endo C, Sakurada A, Hoshi F, Notsuda H, Kondo T. The prognostic significance of eukaryotic elongation factor 1 alpha-2 in non-small cell lung cancer. *Anticancer research.* Feb 2014;34(2):651-658.
95. da Rocha AA, Giorgi RR, de Sa SV, et al. Hepatocyte growth factor-regulated tyrosine kinase substrate (HGS) and guanylate kinase 1 (GUK1) are differentially expressed in GH-secreting adenomas. *Pituitary.* 2006;9(2):83-92.
96. Nishikimi A, Ishihara S, Ozawa M, et al. Rab13 acts downstream of the kinase Mst1 to deliver the integrin LFA-1 to the cell surface for lymphocyte trafficking. *Science signaling.* 2014;7(336):ra72.
97. Ioannou MS, Bell ES, Girard M, et al. DENND2B activates Rab13 at the leading edge of migrating cells and promotes metastatic behavior. *The Journal of cell biology.* Mar 2 2015;208(5):629-648.
98. Wu C, Agrawal S, Vasanji A, et al. Rab13-dependent trafficking of RhoA is required for directional migration and angiogenesis. *The Journal of biological chemistry.* Jul 1 2011;286(26):23511-23520.
99. Kim JH, Lee JY, Lee KT, et al. RGS16 and FosB underexpressed in pancreatic cancer with lymph node metastasis promote tumor progression. *Tumour biology : the journal of the International Society for Oncodevelopmental Biology and Medicine.* Oct 2010;31(5):541-548.
100. Berthebaud M, Riviere C, Jarrier P, et al. RGS16 is a negative regulator of SDF-1-CXCR4 signaling in megakaryocytes. *Blood.* Nov 1 2005;106(9):2962-2968.
101. Lippert E, Yowe DL, Gonzalo JA, et al. Role of regulator of G protein signaling 16 in inflammation-induced T lymphocyte migration and activation. *Journal of immunology.* Aug 1 2003;171(3):1542-1555.
102. Cai W, Ye L, Sun J, Mansel RE, Jiang WG. Expression of MLN64 influences cellular matrix adhesion of breast cancer cells, the role for focal adhesion kinase. *International journal of molecular medicine.* Apr 2010;25(4):573-580.
103. Murcia M, Faraldo-Gomez JD, Maxfield FR, Roux B. Modeling the structure of the StART domains of MLN64 and StAR proteins in complex with cholesterol. *Journal of lipid research.* Dec 2006;47(12):2614-2630.
104. Neilsen PM, Noll JE, Mattiske S, et al. Mutant p53 drives invasion in breast tumors through up-regulation of miR-155. *Oncogene.* Jun 13 2013;32(24):2992-3000.

105. Yao K, Tan J, Ye P, et al. Integrin beta1-mediated signaling is involved in transforming growth factor-beta2-promoted migration in human lens epithelial cells. *Molecular vision.* 2007;13:1769-1776.
106. Zhang C, Zhang F, Tsan R, Fidler IJ. Transforming growth factor-beta2 is a molecular determinant for site-specific melanoma metastasis in the brain. *Cancer research.* Feb 1 2009;69(3):828-835.
107. Miyazawa Y, Uekita T, Ito Y, Seiki M, Yamaguchi H, Sakai R. CDCP1 regulates the function of MT1-MMP and invadopodia-mediated invasion of cancer cells. *Molecular cancer research : MCR.* Jun 2013;11(6):628-637.
108. Uekita T, Fujii S, Miyazawa Y, et al. Suppression of autophagy by CUB domain-containing protein 1 signaling is essential for anchorage-independent survival of lung cancer cells. *Cancer science.* Jul 2013;104(7):865-870.
109. Benes CH, Poulogiannis G, Cantley LC, Soltoff SP. The SRC-associated protein CUB Domain-Containing Protein-1 regulates adhesion and motility. *Oncogene.* Feb 2 2012;31(5):653-663.
110. Ding Z, Jian S, Peng X, et al. Loss of MiR-664 Expression Enhances Cutaneous Malignant Melanoma Proliferation by Upregulating PLP2. *Medicine.* Aug 2015;94(33):e1327.
111. Lee SM, Shin H, Jang SW, et al. PLP2/A4 interacts with CCR1 and stimulates migration of CCR1-expressing HOS cells. *Biochemical and biophysical research communications.* Nov 12 2004;324(2):768-772.
112. Zhu H, Miao MH, Ji XQ, Xue J, Shao XJ. miR-664 negatively regulates PLP2 and promotes cell proliferation and invasion in T-cell acute lymphoblastic leukaemia. *Biochemical and biophysical research communications.* Apr 3 2015;459(2):340-345.
113. Gabrovska PN, Smith RA, Haupt LM, Griffiths LR. Investigation of two Wnt signalling pathway single nucleotide polymorphisms in a breast cancer-affected Australian population. *Twin research and human genetics : the official journal of the International Society for Twin Studies.* Dec 2011;14(6):562-567.
114. Gu W, Pan F, Singer RH. Blocking beta-catenin binding to the ZBP1 promoter represses ZBP1 expression, leading to increased proliferation and migration of metastatic breast-cancer cells. *Journal of cell science.* Jun 1 2009;122(Pt 11):1895-1905.
115. Song T, Zheng Y, Wang Y, et al. Specific interaction of KIF11 with ZBP1 regulates the transport of beta-actin mRNA and cell motility. *Journal of cell science.* Mar 1 2015;128(5):1001-1010.
116. Gu W, Katz Z, Wu B, et al. Regulation of local expression of cell adhesion and motility-related mRNAs in breast cancer cells by IMP1/ZBP1. *Journal of cell science.* Jan 1 2012;125(Pt 1):81-91.
117. Huttelmaier S, Zenklusen D, Lederer M, et al. Spatial regulation of beta-actin translation by Src-dependent phosphorylation of ZBP1. *Nature.* Nov 24 2005;438(7067):512-515.
